# The concept of: Generic drugs and patented drugs vs. brand name drugs and non-proprietary (generic) name drugs

**DOI:** 10.3389/fphar.2013.00113

**Published:** 2013-09-12

**Authors:** Karan B. Thakkar, Gauri Billa

**Affiliations:** ^1^Department of Pharmacology, Grant Government Medical College and Sir J J Group of HospitalsMumbai, India; ^2^Department of Pharmacology, Seth GS Medical College and KEM HospitalMumbai, India

**Keywords:** generic drugs, international non-proprietary name, pharmacoeconomics, generic name, drug costs, bioequivalence studies

Time and again the importance of generic prescribing has been emphasized, primarily to reduce the cost of drugs (Mukherjee, 2013). There are two concepts to be understood here, one is generic vs. patented drugs and the other is a drug's “brand name” vs. “non-proprietary name” or “generic name.” Although, our article primarily describes the Indian scenario, it can be extrapolated to other countries also.

Non-proprietary name is the name for the active ingredient in the medicine that is decided by an expert committee and is understood internationally (WHO, 2013a). Thus, paracetamol/acetaminophen is the non-proprietary name (generic name) while Crocin/Metacin/Meftal/Tylenol etc. are brand names.

It is a well-known fact that generic drugs are “drugs that are usually intended to be interchangeable with an innovator product that is manufactured without a license from the innovator company and marketed after the expiry date of the patent or other exclusive rights” (WHO, 2013b). Bioequivalence is a sine qua non to generic drugs. Good quality bioequivalence studies will help to ensure safety, efficacy, and potency of a generic drug. When it is said that doctors should prescribe generic drugs, it means that they should prescribe drugs manufactured by other companies after expiry of patent of parent drug of the innovator company. Very often, generic prescribing is misconceived as prescribing by a drug's generic name or non-proprietary name. All generic drugs have a brand name as well as a non-proprietary name but all drugs having a non-proprietary name (generic name) may not be generic drugs.

The patent for paracetamol expired in 2007 after which numerous generic versions have been developed and sold under various “brand names.” If one were to prescribe it only by the name “paracetamol” (generic name), it is up to the pharmacist to select and dispense a particular brand, which may either be the costliest brand at ₹ 3.64 (0.06 $) per 500 mg tablet or it could be the cheapest brand at ₹ 0.22 (0.004 $) per 500 mg tablet or somewhere in between (MedGuide India, 2013). A simpler and better alternative for cost reduction would be to prescribe the cheapest “brand” of paracetamol.

This may not be applicable in government drug stores where usually only a single brand of a particular drug is available. In this case, prescribing by generic names is justified because if brand names are used and that particular brand is not available in the drug store, the pharmacist will have to refer drug indexes like CIMS etc. (CIMS—Current Index of Medical Specialties) to find out the ingredient and then dispense from the available stock. This will lead to waste of time and may also lead to errors in case the wrong drug ingredient is dispensed (Cameron et al., 2012). In other countries like the United States of America or the United Kingdom, community pharmacists play an important role in dispensing medicines and hence their cost awareness becomes crucial. But in India, the concept of community pharmacists doesn't exist and hence the onus for cost reduction, from the point of view of drug selection, lies with the doctors.

Thus, a better way to prescribe would be to prescribe the cheapest brand of the drug and include the generic name of the drug in parenthesis, in case that particular brand is not available. For this, the physicians will have to have knowledge about the cost of various brands of a particular drug. It may sound time consuming but in today's internet age, such information is just a click away (MedGuide India, 2013).
